# Malignant Uterine Neoplasms Attended at a Brazilian Regional Hospital: 16-years Profile and Time Elapsed for Diagnosis and Treatment

**DOI:** 10.1055/s-0040-1718434

**Published:** 2021-01-19

**Authors:** Elaine Cristina Candido, Nelio Neves Veiga Junior, Monique Possari Minari, Maria Carolina Szymanski Toledo, Daniela Angerame Yela, Julio Cesar Teixeira

**Affiliations:** 1 Department of Obstetrics and Gynecology, Faculty of Medical Sciences, Universidade Estadual de Campinas, Campinas, SP, Brazil

**Keywords:** endometrial neoplasms, uterine sarcomas, epidemiology, neoplasm staging, survival analysis, neoplasias endometriais, sarcomas uterinos, epidemiologia, estadiamento de neoplasia, análise de sobrevivência

## Abstract

**Objective** The present study aims to evaluate the profile of endometrial carcinomas and uterine sarcomas attended in a Brazilian cancer center in the period from 2001 to 2016 and to analyze the impact of time elapsed from symptoms to diagnoses or treatment in cancer stage and survival.

**Methods** This observational study with 1,190 cases evaluated the year of diagnosis, age-group, cancer stage and histological type. A subgroup of 185 women with endometrioid histology attended in the period from 2012 to 2017 was selected to assess information about initial symptoms, diagnostic methods, overall survival, and to evaluate the influence of the time elapsed from symptoms to diagnosis and treatment on staging and survival. The statistics used were descriptive, trend test, and the Kaplan-Meier method, with *p* -values < 0.05 for significance.

**Results** A total of 1,068 (89.7%) carcinomas (77.2% endometrioid and 22.8% non-endometrioid) and 122 (10.3%) sarcomas were analyzed, with an increasing trend in the period ( *p* < 0.05). Histologies of non-endometrioid carcinomas, G3 endometrioid, and carcinosarcomas constituted 30% of the cases. Non-endometrioid carcinomas and sarcomas were more frequently diagnosed in patients over 70 years of age and those on stage IV ( *p* < 0.05). The endometrioid subgroup with 185 women reported 92% of abnormal uterine bleeding and 43% diagnosis after curettage. The average time elapsed between symptoms to diagnosis was 244 days, and between symptoms to treatment was 376 days, all without association with staging ( *p* = 0.976) and survival ( *p* = 0.160). Only 12% of the patients started treatment up to 60 days after diagnosis.

**Conclusion** The number of uterine carcinoma and sarcoma cases increased over the period of 2001 to 2016. Aggressive histology comprised 30% of the patients and, for endometrioid carcinomas, the time elapsed between symptoms and diagnosis or treatment was long, although without association with staging or survival.

## Resumo

**Objetivo** O presente estudo avaliou o perfil dos carcinomas endometriais e sarcomas uterinos atendidos em um centro brasileiro de câncer no período de 2001 a 2016, e avaliou o impacto do tempo decorrido entre os sintomas até o diagnóstico ou tratamento no estadiamento e sobrevida pelo câncer.

**Métodos** Estudo observacional com 1.190 casos que analisou o ano do diagnóstico, faixa etária, estágio e tipo histológico do câncer. Um subgrupo de 185 mulheres com histologia endometrioide e atendidas no período de 2012 a 2017 foi selecionado para avaliar informações sobre sintomas iniciais, métodos de diagnóstico, sobrevida global e para analisar a relação entre o tempo decorrido a partir dos sintomas até o diagnóstico e tratamento no estadiamento e sobrevida. Foram realizadas análises estatísticas descritiva, de tendência linear e de sobrevida pelo método de Kaplan-Meier, com valores de *p* < 0,05 para significância.

**Resultados** Os casos estudados de acordo com a histologia foram 1.068 (89,7%) carcinomas (77,2% endometrioides e 22,8% não endometrioides) e 122 (10,3%) sarcomas, com tendência crescente no período ( *p* < 0,05). Histologias de carcinomas não endometrioides, G3 endometrioides e carcinossarcomas consistiram em 30% dos casos. Carcinomas não endometrioides e sarcomas foram mais frequentemente diagnosticados em pacientes acima de 70 anos de idade e em estágio IV ( *p* < 0,05). O subgrupo com 185 mulheres com carcinoma endometrioide apresentou 92% de sangramento uterino anormal e 43% de diagnóstico após curetagem. O tempo médio decorrido entre os sintomas e o diagnóstico foi de 244 dias e entre os sintomas e o tratamento, 376 dias, todos sem associação com estadiamento ( *p* = 0,976) e sobrevida ( *p* = 0,160). Apenas 12% das pacientes iniciaram o tratamento em até 60 dias após o diagnóstico.

**Conclusão** O número de casos de carcinomas e sarcomas uterinos aumentaram no período de 2001 a 2016. A histologia agressiva compreendeu 30% dos pacientes e, no caso dos carcinomas endometrioides, o tempo decorrido entre os sintomas e o diagnóstico ou tratamento foi longo, embora sem associação com estadiamento ou sobrevida.

## Introduction

Malignant uterine neoplasms are the most common type of gynecological cancers worldwide. Among the most frequent neoplasms, uterine cancer occupies the 6 ^th^ position, with ∼ 380 thousand new cases per year. Its incidence has increased in recent decades, especially in developed countries. 1 In Brazil, the estimated incidence for 2020 is 6,540 new cases, with a higher expected incidence in the southeast region. 2

These neoplasms are divided into two major categories: carcinomas and sarcomas. Endometrial carcinomas are responsible for ∼ 95% of all uterine malignancies. 3 4 5 Risk factors for carcinomas are obesity, sedentary lifestyle, and increased life expectancy, which are factors related to a higher Human Development Index. 6 7 Conversely, regular physical activity and proper use of hormonal therapies (estrogens associated with progestogens) can reduce the risk. 6 7 In relation to uterine sarcomas, there is little information about risk factors. However, some studies show that advanced age and previous radiotherapy can be associated with sarcoma development. 4 5 8

Staging and tumor histology are considered the main prognostic factors of malignant uterine neoplasms, with better prognosis described for endometrioid carcinomas. On the other hand, poorly differentiated or non-endometrioid carcinomas and sarcomas have a worse prognosis. 9 10 The gap between first symptoms and diagnosis or treatment onset can modify the staging and therapeutic results, mainly for worse prognosis cases. In less developed countries or regions with difficult access to health care, it is common to have delays in scheduling appointments, diagnostic investigation, and referral to oncologic centers. However, this delay may be less relevant in neoplasms with slow evolution or a good prognosis.

The present study aims to obtain information about the diagnosis profile of malignant uterine neoplasms diagnosed in women assisted in the public system of a regional cancer center in Brazil, and the influence of the time elapsed between the first symptoms to diagnosis or treatment in staging and survival of endometrioid carcinomas.

## Methods

An observational study was performed based on retrospective data extracted from the Hospital-Based Cancer Registries (HCR) system of the Women's Health Hospital of the University of Campinas, from 2001 to 2016. We identified 1,243 records filtered by code C.54 (uterine body malignant neoplasia) from the International Classification of Diseases, 10th edition (ICD-10). 11 The included cases were from 90 cities (6.8 million inhabitants) that make up the Administrative Region of Campinas (São Paulo, Brazil). 12 A total of 51 cases from other cities, one case with two synchronous neoplasms and one cervix cancer case were excluded, resulting in 1,190 cases. Information was collected regarding the year of diagnosis, age, histological type, and staging according to the International Federation of Gynecology and Obstetrics (FIGO, 2009 for sarcomas and 2014 for carcinomas). 13 14 Tumor histology was defined according to the pathologist's final report and the International Histological Classification of Tumors of the World Health Organization. 15 The main categories of carcinomas were endometrioid types (or Bokhman type I), non-endometrioid (or Bokhman type II, including serous, serous-papillary, clear-cell, squamous-cell, mucinous, neuroendocrine or mixed histologies), and the poorly-differentiated or undifferentiated ones. Among neoplasms with a sarcomatous component (also called ‘sarcomas’), we found carcinosarcomas (including Mullerian tumors and heterologous sarcomas), leiomyosarcomas, endometrial stromal sarcomas, and adenosarcomas. 16 Thereafter, a subgroup with 185 women of up to 85 years of age, with endometrioid histology and who had been attended from June 2012 to June 2017, was selected to assess the association between the time elapsed from the onset of symptoms until diagnosis or treatment and the cancer stage and survival. The selection considered women from the region covered by the Regional Health Board VII - Campinas (42 cities and 4.43 million people). 17 All of the 185 women had their medical records reviewed, and information was collected about initial symptoms, diagnostic methods, and posttreatment outcomes, and the interval between symptoms and diagnosis and between symptoms and treatment onset was calculated.

### Statistical Analysis

The rates of diagnoses recorded between 2001 and 2016 were analyzed according to the biennial period, age group (< 50 years, 50–59, 60–69 years and > 70 years), staging (I–IV) and by histological types grouped into carcinomas *versus* (versus) sarcomas and endometrioid carcinomas (including the histological differentiation degree-G) versus non-endometrioid. The Chi-square, linear trend and Fisher tests were used. Treatment outcomes were evaluated by Kaplan-Meier survival analyses and the Log-rank test. Statistical analysis was made using the StatsDirect statistical software v. 3.0 (StatsDirect, Cheshire, United Kingdom), and *p* -values of less than 0.05 were considered statistically significant.

This study followed the recommendations of the National Health Council of Brazil and was previously approved by the Ethics Committee of the University of Campinas (Certificate of Ethical Assessment CAAE 48055015.3.0000.5404; approval number 1.760.085, October 4, 2016).

## Results

A total of 1,068 (89.7%) carcinomas (77.2% endometrioid and 22.8% non-endometrioid) and 122 (10.3%) sarcomas ( Table 1 ) were analyzed. Considering only carcinomas with worse prognosis, such as non-endometrioid carcinomas, endometrioid G3, and carcinosarcomas, we had 354 cases, or 29.8%.

**Table 1:** Distribution of the histological types of 1,190 malignant uterine neoplasms

Histological types	n	%
Carcinomas	1,068	89.7
Endometrioid	825	77.2
Grade * 1	(286)	(34.7)
Grade 2	(485)	(58.8)
Grade 3	(54)	(6.5)
Non endometrioid	243	22.8
Serous or papillary	(90)	(37.0)
Clear cells	(48)	(19.8)
Mucinous	(24)	(9.9)
Squamous cell	(9)	(3.7)
Poor or undifferentiated	(27)	(11.1)
Neuroendocrine	(3)	(1.2)
Mixed	(39)	(16.1)
Other	(3)	(1.2)
Sarcomas	122	10.3
Carcinosarcoma **	(57)	(46.7)
Leiomyosarcoma	(27)	(22.1)
Endometrial stromal sarcoma	(20)	(16.5)
Adenosarcoma	(17)	(13.9)
Other (rhabdomyosarcoma)	(1)	(0.8)

* Degree of histological differentiation.

** Carcinosarcomas were considered together with sarcomas, although they follow the staging of carcinomas.

**Fig. 1:**
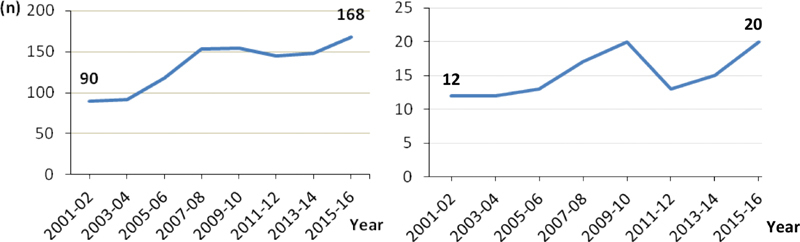
Biennial distribution of malignant uterine neoplasms according to the histological group, carcinomas ( *n* = 1,068), or sarcomas (including carcinosarcomas) ( *n* = 122).

A trend toward increasing cases was registered in the evaluated period for carcinomas and sarcomas, with an additional 10.8 cases of carcinomas ( *p* = 0.003) and 1.2 cases of sarcomas ( *p* = 0.044) every 2 years. Between 2001 and 2002, 90 carcinomas and 12 sarcomas were recorded, and this number increased to 168 carcinomas and 20 sarcomas for the 2015 to 2016 biennium ( Fig. 1 ). The carcinomas and sarcomas diagnoses' proportions remained constant in the evaluated period, around 90% of carcinomas and 10% of sarcomas.

The mean age of the patients was 62 years, similar between carcinomas and sarcomas. Table 2 shows the diagnoses distribution by age group, with 61 to 63% of the cases occurring in women aged 60 or over. Sarcomas were more diagnosed in the extreme age groups, such as under 50 years old (16.4% versus 8.4% for carcinomas) and 70 years old or more (34.4% versus 25.7% for carcinomas) ( *p* = 0.001). Concerning cancer stage, stage I was mostly found in carcinomas (57.7% versus 42.2%) and stage IV (21.3% versus 7.1%) was 3-fold more frequent in sarcomas ( *p* < 0.0001).

**Table 2:** Distribution of malignant uterine neoplasms diagnosed in the period from 2001 to 2016 according to age group and stage

Variable	Carcinomas ( *n* = 1,068)		Sarcomas ( *n* = 122)		Total ( *n* = 1,190)	
	n	%	n	%	n	%
Age group (years)						
< 50	90	8.4	20	16.4	110	9.3
50–59	302	28.3	27	22.1	329	27.7
60–69	402	37.6	33	27.1	435	36.5
≥70	274	25.7	42	34.4	316	26.5
Stage *						
I	615	57.7	54	42.2	669	56.2
II	126	11.8	13	10.7	139	11.7
III	250	23.4	29	23.8	279	23.5
IV	76	7.1	26	21.3	102	8.6
*Miss information*	1				1	

Chi-square test: Age-group ( *p* = 0.001); Stage ( *p* < 0.0001).

* Staging system according to FIGO-2014 for carcinomas and FIGO-2009 for sarcomas.

**Fig. 2:**
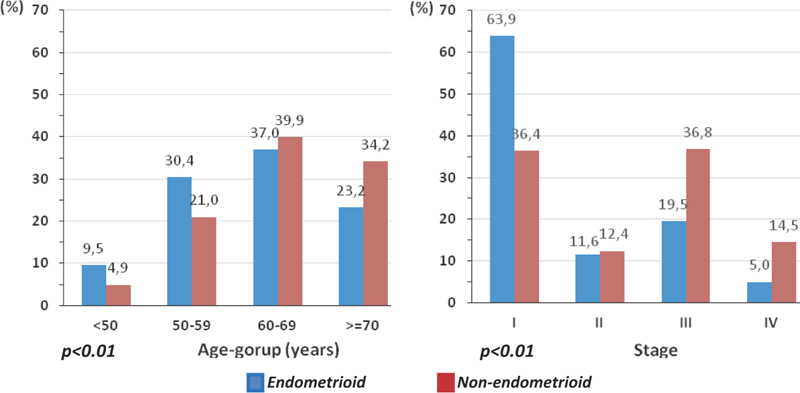
Distribution of uterine carcinomas by histology endometrioid ( *n* = 825) and non-endometrioid ( *n* = 243) according to age-group and neoplasia stage (FIGO-2014; there is no stage information for one case).

Between endometrioid carcinomas versus non-endometrioid, significant differences were observed in the mean ages (62.1 + 10.3 years versus 65.6 + 10.2 years, respectively, *p* < 0.001), and in the distributions by age groups or by staging ( Fig. 2 ). Endometrioid carcinomas occurred in a greater proportion of younger age groups and stage I compared with non-endometrioid types, which occurred in older patients and was diagnosed more often in stages II to IV ( *p* < 0.01).

The evaluation of the 185 women subgroup with endometrioid carcinoma exhibited a mean age (65.5 years; 54–83) and staging distribution (stage I = 65.9%) similar to the main group ( *p* > 0.05), but with more cases of G3 neoplasms (G3 = 22 or 11.9% versus 6.6%, *p* = 0.003; G1 = 44 or 24% and G2 = 119 or 64%). Overall five-year survival rate was 76.9%, better for stage I (93.8% versus 43.0% for stages II–IV, *p* < 0.0001), and significantly worse for G3 carcinomas (49.9% versus 77.1% for G2 and 89.4% for G1, *p* = 0.012). Table 3 describes the symptom's pattern, diagnostic method, and time elapsed to diagnosis and treatment for the 185-women subgroup. Three patients did not receive treatment for advanced disease and died early. Abnormal uterine bleeding was highlighted in 92.4% of the cases (as a unique symptom in 87.0% of the cases). Uterine curettage was the most used diagnostic method (43.2%), followed by hysteroscopy (36.2%) and endometrial biopsy (17.9%). The mean time elapsed between the onset of symptoms (or suspicion) and the cancer diagnosis was 244 days (24.3% up to 90 days, 25.6% between 91 and 180 days, 30.3% between 181 and 365 days, and 19.5% more than 365 days).

**Table 3:** Pattern of symptoms, diagnostic method, and time elapsed to diagnosis and treatment in 185 women with endometrioid carcinomas

Characteristic	n	%
Symptom (sign) at diagnosis *		
Uterine abnormal bleeding	171	92.4
Endometrial thickening (ultrasound)	7	3.8
Vaginal discharge	4	2.2
Pelvic pain	2	1.1
Abnormal clinical exam	1	0.5
Diagnostic method		
Dilation and curettage	80	43.2
Hysteroscopy	67	36.2
Aspiration biopsy	33	17.9
Hysterectomy	5	2.7
Symptom to diagnosis time (days)		
Mean (SD)	244 (±44)	
≤ 90	45	24.3
91–180	48	25.9
181–365	56	30.3
> 365	36	19.5
Diagnosis to treatment time (days) **		
Mean (SD)	131 (±71)	
≤ 60	22	12.1
61–90	17	9.3
> 90	143	78.6
Symptom to treatment time (days) **		
Mean (SD)	376 (±49)	
≤ 180	33	18.1
181–365	72	39.6
> 365	77	42.3

Abbreviation: SD, standard deviation.

* considered only the main one.

** Three patients did not receive treatment for advanced disease and were early dead.

There was no association between the time elapsed and the final cancer stage ( *p* = 0.976) ( Table 4 ).

**Table 4:** The final stage according to the time elapsed (days) from symptoms to diagnosis in 185 women with endometrioid carcinomas

Stage *	Symptom to diagnosis time (days)							
	≤ 90		91–180		181–365		> 365	
	n	%	n	%	n	%	n	%
I	27	60.0	33	68.7	38	67.8	24	66.7
II	7	15.6	7	14.6	8	14.3	5	13.9
III and IV	11	24.4	8	16.7	10	17.9	7	19.4
Total	45	100	48	100	56	100	36	100

* Staging system according to FIGO-2014; *p* = 0.976 (Chi-square test).

**Fig. 3:**
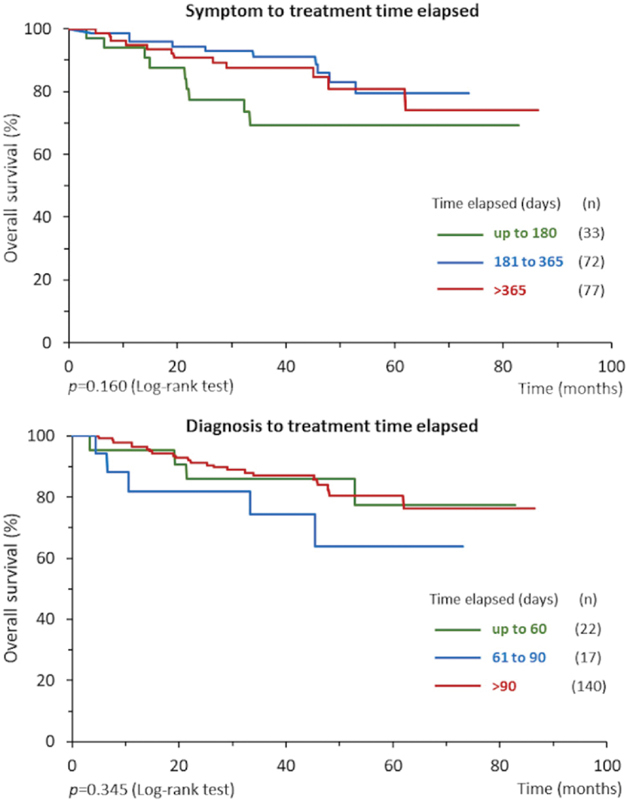
Overall survival of 182 women with endometrioid carcinomas according to time elapsed (days) from symptoms (or suspicion) to start treatment (upper chart), and from diagnosis to start treatment (bottom chart).

Among the 182 patients treated, the mean time elapsed between the onset of symptoms (or suspicion) and onset of treatment was 376 days, with 81.9% of the cases taking more than 180 days, and there was no association between time elapsed and overall survival rate ( Fig. 3 , *p* = 0.160). The mean time interval between diagnosis and treatment onset was 131 days, with only 12.1% of the patients having started treatment within 60 days of diagnosis (legal deadline according to current Brazilian legislation). There was no association between this time elapsed and the overall survival rate ( *p* = 0.345), as shown in the ( Fig. 3 ) bottom chart.

## Discussion

The malignant uterine neoplasms new cases, including carcinomas and sarcomas, increased throughout th period from 2001 to 2016. A rate of 90% of carcinomas and 10% of sarcomas was maintained in the period, with 60% of all cases aged 60 years or more. Among the carcinomas, 77% were endometrioid type, and the most frequent sarcomas diagnosed were carcinosarcomas, with 46.7%, followed by leiomyosarcomas (22.1%) and endometrial stromal sarcomas (16.4%). Sarcomas were 3-fold more diagnosed in stage IV (disseminated disease).

The upward trend in the new cases attended of malignant uterine neoplasms may be an effect of population growth, increased life expectancy, and due to greater request for the unified health system ( *Sistema Único de Saúde* – SUS, in Portuguese), as observed in times of economic crisis. The Women's Health Hospital is the main cancer center for gynecological oncologic care for a population of 6.5 million people, with 50% of SUS users. 17 Notedly, for endometrioid carcinomas, obesity is considered an important risk factor 6 7 and this condition is gradually more frequent and associated with developed areas, as well as the region where these cases came from.

The increase in the number of new cases of malignant uterine neoplasms differs from American statistics for the period of 2005 to 2014, according to which the rates have not changed significantly, 4 perhaps due to stable epidemiological factors for a longer time.

As per the analysis by age group, these malignant uterine neoplasms predominated in the group above 60 years of age, with 34% of sarcomas and 46.7% of carcinosarcomas being diagnosed at 70 years old, highlighting the classic association between aging and cancer. 18 However, it is worth noting that 110 women (9.3%) presenting some of these neoplasms were under 50 years old, which was twice more frequent in the sarcomas' group (16.4% versus 8.4%, *p* = 0.001). Among all 20 cases of sarcomas under 50 years of age, 9 were endometrial stromal sarcomas and 7 were leiomyosarcomas, which are estrogen-related neoplasms of younger women. No carcinosarcoma cases were observed in this age group since they are related to elderly women. 5 19

Usually, carcinomas tend to be symptomatic earlier than sarcomas, and sarcomas tend to have a faster and more aggressive progression. Thereafter, and according to our results, carcinomas were detected in a greater proportion in stage I (57.7% versus 42.2%) and sarcomas were 3-fold more frequently diagnosed in stage IV (21.3% versus 7.1%, *p* < 0.0001).

Comparing endometrioid and non-endometrioid carcinomas, the etiological factors must be considered. The endometrioid histology is related to endometrial hyperplasia due to the estrogenic effect, supporting an earlier diagnosis and better prognosis with a 5-year survival rate of 85%. 16 20 21 Relatively, non-endometrioid carcinomas affect older women, are associated with atrophic endometrium, high nuclear grade, and poor cell differentiation, resulting in earlier myometrial infiltration and lymph node involvement, with shorter 5-year survival around 59%. 16 20 21

Our results confirmed some differences between endometrioid and non-endometrioid carcinomas, such as twice as many endometrioid carcinomas in patients under 50 years old (9.5% *vs* . 4.9%) and 34% of non-endometrioid recorded in women aged 70 and over. Stage I prevailed among endometrioid cases (63.9%), while there were 3-fold more stage IV non-endometrioid carcinomas (14.5% *vs* . 5%) ( *p* < 0.01).

Analyzing the group of 185 women with endometrioid carcinomas, 92.4% of them exhibited genital abnormal bleeding. Diagnosis accessing by hysteroscopy (36.2%) or outpatient endometrial biopsy (17.9%) were not predominant. The mean time elapsed from symptoms to diagnosis can be considered long, 244 days, with 75.7% of cases taking more than 90 days. Furthermore, the mean interval from symptoms to treatment was longer and concerning, 376 days, with 81.8% over 180 days.

The long periods observed waiting for a diagnosis can be associated with several factors, such as the lack of qualified gynecological care, the difficulty of accessing investigation methods, and the lack of guidelines for the management of suspected cases. This shows, in practice, the inefficient assistance of the secondary level of the public health system in Brazil. Also, the cultural level and resignation of most SUS users, associated with a certain technical limitation of health professionals, contribute to this scenario. And, lastly, difficulties continue for diagnosed neoplasia due to limited assistance offered in the oncology centers of SUS.

Although the present study confirms the long waiting time for diagnosis and to start treatment, surprisingly and unexpectedly, there was no negative impact on the final staging or overall survival. This may be related to the fact that endometrioid carcinomas have a better prognosis. This histological type was analyzed in detail because they comprise a greater proportion of cases, with early symptoms and better prognosis, that is, cases in which the health care system must not fail. The time elapsed between symptoms and diagnosis was longer than diagnosis and treatment, pointing out the difficulties related to the current primary and secondary level of care in the public health system. We planned to start a new study to evaluate the relationship between the time elapsed for diagnoses and non-endometrioid histology.

After the diagnosis, the limitation of the public system in providing care for women with gynecological cancer is clear, as previously mentioned by other Brazilian authors, 22 even though the cases studied come from a high human development index area. We noticed that only 12.1% of women were able to start treatment within 60 days after diagnosis, as determined by Brazilian Federal Law 12,732/12, valid since 23/05/2013. 23 About 80% of the cases waited for treatment for more than 90 days, confirming a deficiency in complying with the law. Although not shown in the results, most of the time elapsed between diagnosis and treatment onset occurred before the patients arrived at the cancer center, which, despite being overloaded, usually manages to start the treatment available relatively quickly.

Among the limitations of the present study, using a single institution as origin of the cases can lead to higher proportions of more aggressive histological types. In the literature, the expected rate would be 3 to 5% for sarcomas and 15% for type-II carcinomas. 3 4 5 24 However, we found 10% of sarcomas and 23% of non-endometrioid carcinomas. This situation is justified, in part, by the frequent management of endometrioid carcinomas by general gynecologists since initial stages exhibit better prognosis. Similar to our results, the literature shows that around 70% of the carcinomas are endometrioid type and a 5-year overall survival rate of 90% for stage I cases. 24

Conversely, the strength of the present study is the qualified information retrieved from HCR (accounts for 100% of the cases attended and with periodic review of information) and the fact that this institution is a reference center in gynecological cancer care with a specialized multidisciplinary team. It is also the main reference for cancer care of SUS, in a wealthy developed area. Such characteristics reflect the relevance of the data analyzed related as they show real-life scenarios. This study provides important information that allows a review of assistance programs offered to the population. The adequate protocol development to care for symptomatic postmenopausal women, stratified by levels of complexity, with accurate diagnosis methods, may shorten the waiting time to treatment onset. It is worth noting that 29.8% of the cases studied were considered to have a worse prognosis, and the impact of waiting time for them was not analyzed.

## Conclusion

In conclusion, the malignant uterine neoplasms occurrence increased over the period from 2001 to 2016, for both carcinomas and sarcomas. There are fewer cases of non-endometrioid carcinomas and sarcomas, which are more aggressive and present in higher proportion among women over 70 years old and in stage IV. For endometrioid carcinomas, the time elapsed between symptoms and diagnosis or beginning of treatment was long, although without association with worsening of staging or survival.
